# Management of an Infected Nonunion of an Opening-Wedge High Tibial Osteotomy with 2-Stage Implantation of Rotating Hinge Knee Prosthesis

**DOI:** 10.1155/2018/2493095

**Published:** 2018-01-31

**Authors:** Sandrine Mariaux, Olivier Borens

**Affiliations:** Service of Orthopaedics and Traumatology, Lausanne University Hospital, Lausanne, Switzerland

## Abstract

**Introduction:**

High tibial osteotomy (HTO) is a frequent and effective treatment for unicompartmental gonarthritis. Only a few articles are focused on the treatment of infected nonunion.

**Patient and Method:**

A 50-year-old obese patient was operated on by medial opening-wedge HTO. She developed a painful nonunion treated by hardware removal, allograft, and plate fixation. However, the nonunion persisted. 2 years later, cellulitis appeared with an abscess adjacent to the HTO plate. Despite surgical debridement and antibiotics, septic knee arthritis occurred. In a situation of infected nonunion and septic arthritis with chondrolysis, she was scheduled for a 2-stage total knee replacement (TKR). The infected tibial articular block was first resected and replaced by a cement spacer. After a short interval, the TKR was implanted. After 2 years, the patient walked pain-free with good knee function.

**Discussion:**

In the literature, different efficient treatments exist for infected nonunion after HTO, but comprehensive studies are missing for a consensus treatment. Current data are mostly based on case reports, since this pathology is quite rare.

**Conclusion:**

In a difficult situation of infected nonunion with septic knee arthritis, we performed a 2-stage knee prosthesis implantation. This led to an early mobilization and fast recovery.

## 1. Introduction

High tibial osteotomy (HTO) is an effective treatment for unicompartmental gonarthritis [1]. While it is assumed that opening-wedge high tibial osteotomy has a higher rate of nonunion compared to closed-wedge HTO [2], an equivalent risk of infection and nonunion exists for both techniques [3–5]. Although many studies are found in the literature concerning HTO, only a few papers focus on treatment of infected nonunion [6–8].

## 2. Patient and Method

**Figure 1:**
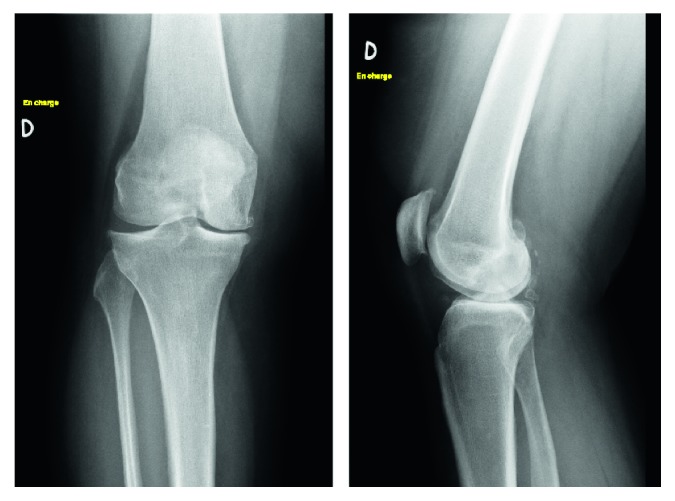
Preoperative weight-bearing X-rays showing narrowing of the joint space in the internal femorotibial compartment.

**Figure 2:**
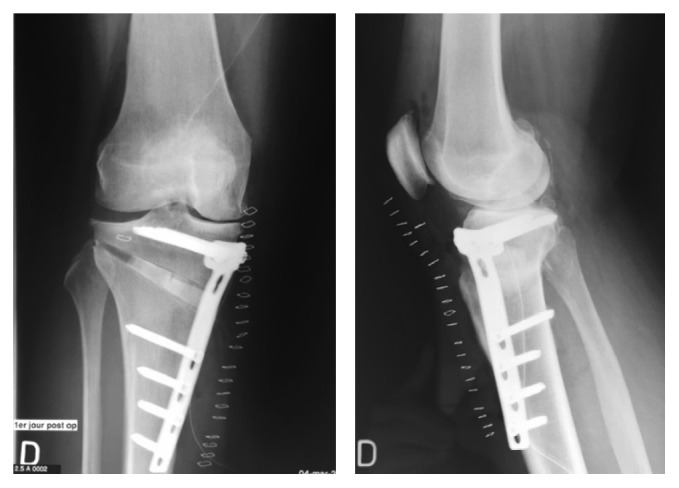
Postoperative X-rays showing fracture of the lateral cortex at the osteotomy site.

**Figure 3:**
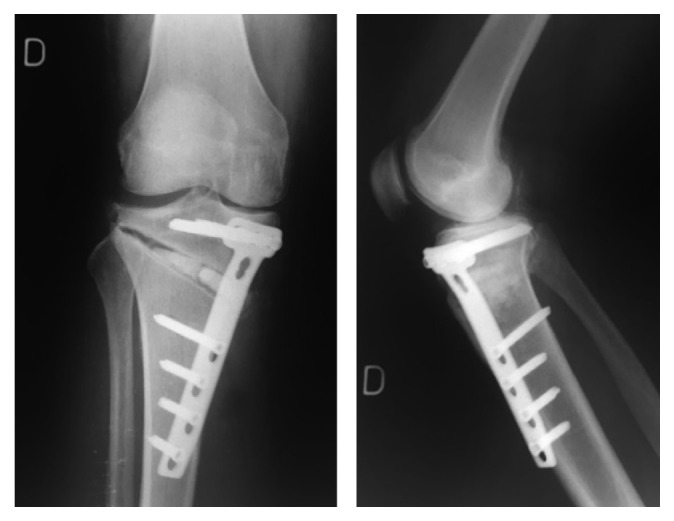
June 2009, 3 months after HTO, postoperative X-rays showing a slight pullout of the proximal screws. No fracture of the plate. No translation of the proximal fragment of the HTO.

**Figure 4:**
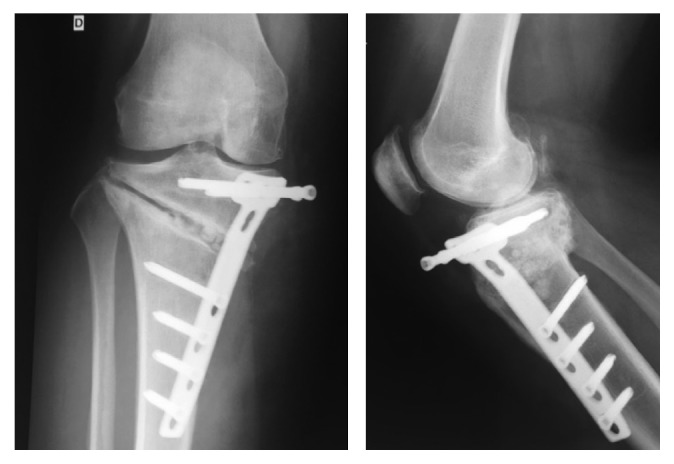
Early June 2010, 1 year after HTO, postoperative X-rays showing an important pullout of the proximal screws and slight translation of the proximal fragment of the HTO.

A 50-year-old female obese patient (BMI 41) known for early varus gonarthritis (Figure 1) was operated on by medial opening-wedge HTO with a TomoFix® plate (DePuySynthes, USA) and osteoconductive tricalcium phosphate resorbable bone substitute (chronOS®, DePuySynthes, USA) in March 2009 (Figure 2). The varus was measured at 5° preoperatively. An arthroscopy done at the beginning of the surgery confirmed a grade II chondropathy of the medial femorotibial compartment based on Outerbridge classification with intact lateral femorotibial or femoropatellar compartments. Early postoperative infection with swelling, redness, and oozing of the wound was treated at the initial hospital with empiric oral co-amoxicillin for two weeks. The wound healed and the evolution was initially favorable. At 3 months postoperatively, the patient complained of light persistent dull pain when walking and X-rays showed a slight pullout of the proximal screws (Figure 3), increasing at 1 year postoperatively (Figure 4).

**Figure 5:**
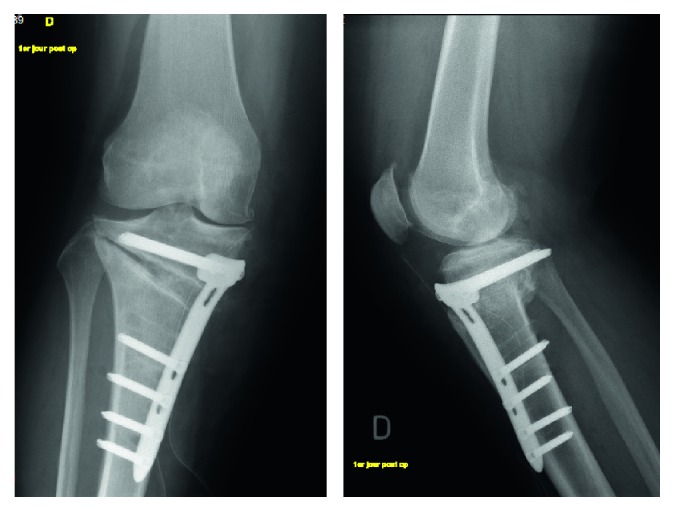
June 2010, postoperative X-rays after revision.

In June 2010, the patient was diagnosed with painful nonunion of the HTO, treated by hardware removal, implantation of osteoinductive demineralized bone matrix (DBX®, DePuySynthes, USA) and osteoconductive allograft (bone chips by Musculoskeletal Transplant Foundation, MTF® DePuySynthes, USA), and fixation with a TomoFix plate (DePuySynthes, USA) (Figure 5). Preoperatively, the wound was calm. No preoperative imaging (scintigraphy, MRI) was done in order to exclude a low-grade infection. Intraoperatively important metallosis and no sign of infection were observed. Unfortunately, no microbiological work up (like sonication of osteosynthesic material or tissue sampling) was performed at that moment. The fact that the early postoperative infection after the first operation had evolved favorably with oral antibiotics in 2009 was considered enough to exclude infection.

**Figure 6:**
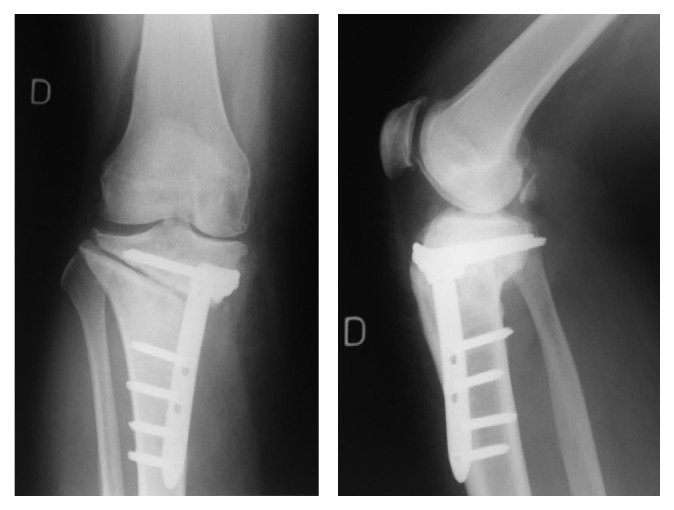
June 2011, 1-year after revision, postoperative X-rays showing fracture of the plate and translation of the proximal fragment of the HTO.

**Figure 7:**
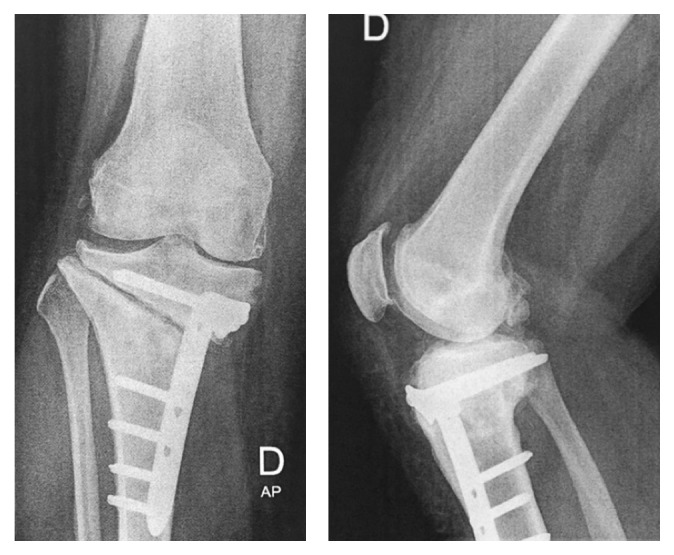
June 2012, 2 years after revision, postoperative X-rays showing medial gonarthritis.

The clinical evolution was satisfactory, and no wound complication occurred. At the 1-year follow-up, X-rays showed persistence of the pseudarthrosis with a broken plate and slight epiphyseal translation (Figure 6). As the patient was asymptomatic, she refused a new operation. 2 years after revision, the X-rays showed a narrowing of the medial femorotibial compartment (Figure 7).

**Figure 8:**
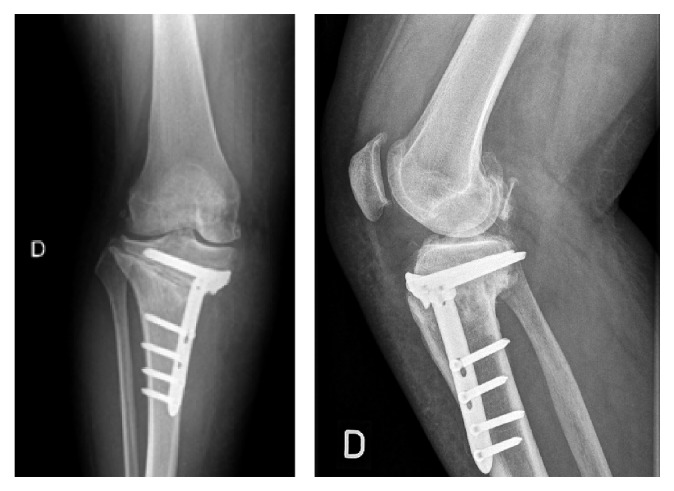
July 2013, 3 years after revision, postoperative X-rays showing increasing signs of osteoarthritis.

In late August 2013, she developed redness and swelling of the right lower extremity, with impossibility to bear weight. A superficial skin infection was suspected, and she was initially hospitalized in the internal medicine unit and treated with intravenous co-amoxicillin. Due to poor local evolution, an ultrasound examination was done, confirming deep wound infection with abscess formation adjacent to the HTO plate. Standard X-rays showed an early bicompartmental gonarthritis (Figure 8). The patient was transferred to the surgery unit, and debridement and hardware removal were performed with ongoing intravenous co-amoxicillin. All tissue samples remained sterile, but molecular analysis by polymerase chain reaction (PCR) revealed *Streptococcus dysgalactiae*. After two more debridements and vacuum-assisted closure dressing (V.A.C.® by KCI, Acelity Company), the clinical course was satisfactory and the wound was finally closed. IV antibiotics were switched to oral levofloxacin in accordance with the resistance pattern of the germ.

**Figure 9:**
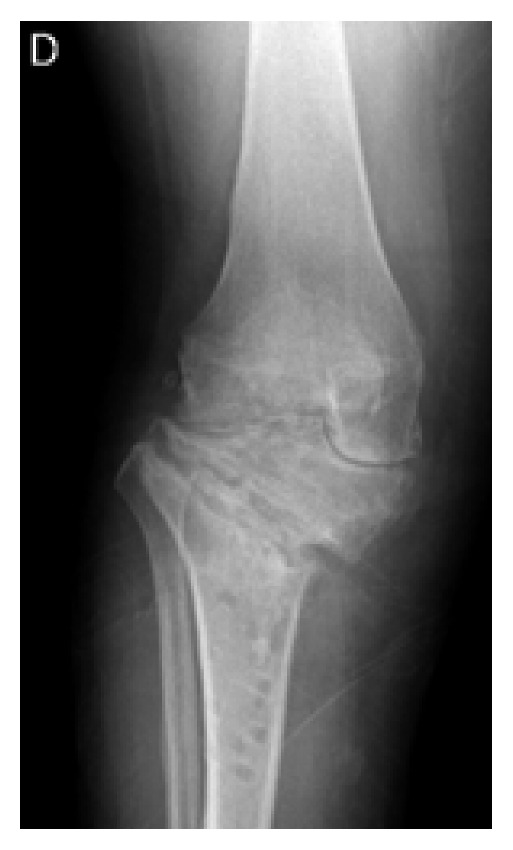
September 2013, after multiple debridements and arthroscopic washout and synovectomy, X-ray showing severe gonarthritis.

Two weeks later, the patient complained of increasing knee pain. Elevated C-reactive protein (CRP) associated with appearance of a new collection next to the wound with knee effusion was found. A CT scan showed a purulent collection around the nonunion with knee effusion. A knee puncture produced a translucent, bloody liquid, which remained sterile under ongoing oral antibiotics. Although the knee puncture remained sterile, considering the clinical status, septic arthritis of the knee was suspected. Open debridement of the proximal tibia was performed, combined with arthroscopic washout and synovectomy. At this stage, X-rays showed an increase of the knee arthritis (Figure 9). The wound was again covered with a V.A.C. dressing, and antibiotics were switched to intravenous piperacillin/tazobactam.

**Figure 10:**
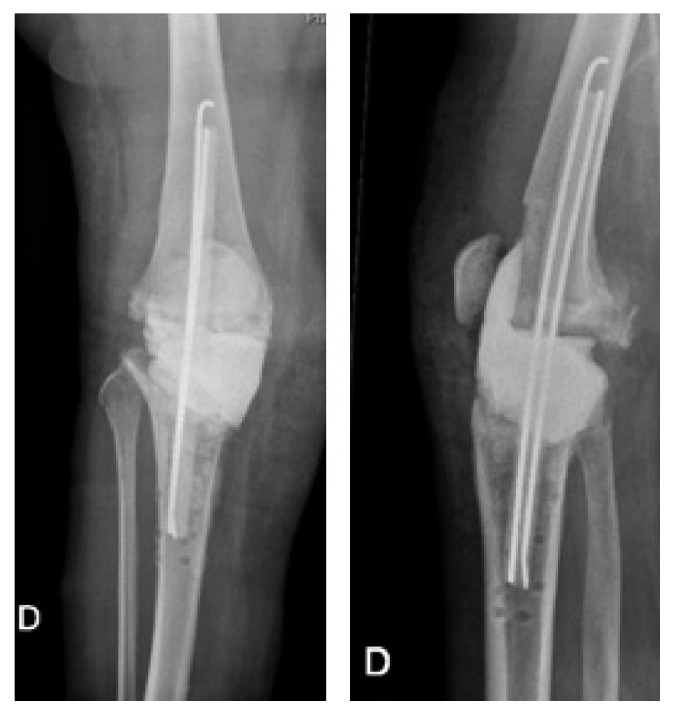
September 2013, X-rays showing postarticular resection and implantation of cement spacer.

Due to a poor outcome, the patient was transferred to our tertiary care hospital, where we decided to opt for a 2-stage operation. Firstly, the partially necrotic, infected tibial articular block was resected (as well as the articular part of the distal femur) and then replaced by a nonarticulating, gentamicin/vancomycin-impregnated polymethylmethacrylate (PMMA)-cement spacer (Figure 10). At this stage, primary closure of the wound was possible. The operation was performed 5 weeks after the initial debridement. Postoperatively, the patient received intravenous co-amoxicillin during 14 days, which was then switched to intravenous penicillin until the prosthesis implantation.

**Figure 11:**
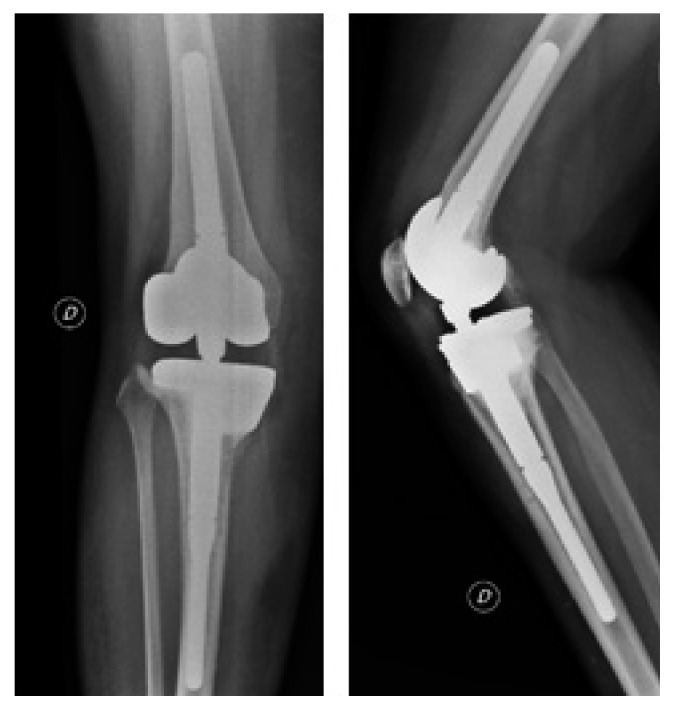
X-rays showing 3 months postoperatively rotating hinge total knee replacement with metal augment.

After 3 weeks and without an antibiotic-free interval, a rotating hinge knee (RHK) prosthesis (Zimmer NexGen Rotating Hinge Knee Revision® with augments) was implanted. Due to necrotic tissue on the edge of the wound and prosthesis volume, a gastrocnemius muscle flap to cover the soft tissue was performed (Figure 11). The RHK prosthesis was chosen due to the important bone loss and the associated ligamentous deficiency. The keels allowed the distribution of the load of the prosthesis to avoid early mechanical failure or loosening. Bone biopsies and sonication of the spacer remained sterile. Once these results were obtained, the patient was switched to oral amoxicillin to complete a total of 3 months of antibiotics.

**Figure 12:**
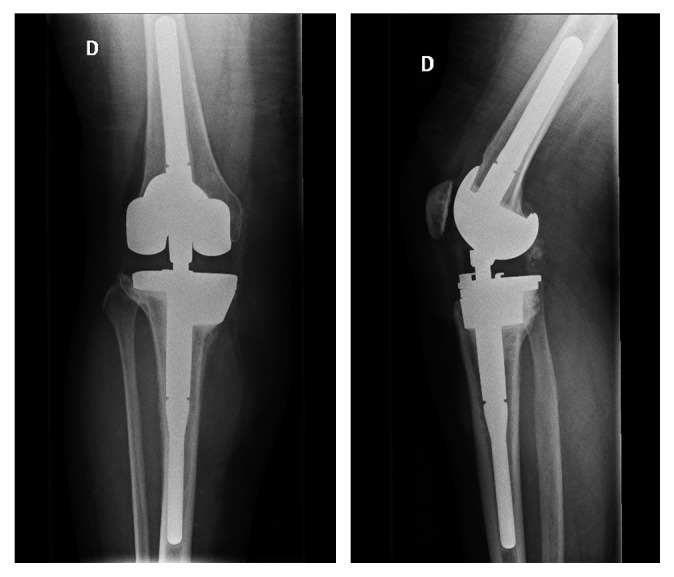
X-rays showing 2 years postoperatively rotating hinge total knee replacement with metal augment.

Immediately after operation, the patient was allowed to walk, bearing their full weight. At the 2-year follow-up, she walked pain-free and had a function of 110-0-0 of the knee (Figure 12). The wound was completely healed, with no complication after muscle flap. No functional score was done postoperatively.

## 3. Discussion

We have presented an infected nonunion after HTO. Our case shows that an infected HTO can be complicated by septic arthritis of the knee, especially when there is a break of the lateral cortex of the proximal tibia.

We could argue about the initial operative indication of HTO in a patient with a BMI over 30. However, Floerkemeier et al. in a study of 386 patients with opening-wedge HTO using locking plate (TomoFix plate, DePuySynthes, USA), concluded that patients with BMI over 30 had a slightly decreased functional outcome but no difference in the complication rate, compared to patients with BMI ≤ 30 [13]. Other authors agree with these results and confirm the higher risk of loss of correction in patients with BMI over 32.5 [12, 14]. So, our patient had a higher risk of complication but had no proven contraindication for this procedure.

In the initial HTO and its revision, allograft was chosen to avoid local complications on the donor site in an obese patient (infection, haematoma, fracture, wound complication, and pain) [9]. In the literature, the correlation between delayed union and allograft is not completely solved. A systematic review including over 3000 patients showed a significant difference in the rate of delayed union and nonunion between autograft (2.6%) and allograft (4.5%). Nevertheless, this review includes 56 different studies with other confounding factors (e.g., the type of plates). Other retrospective studies did not confirm this significant difference [10–12]. Concerning the allograft, calcium phosphate has the highest compressive strength. Tricalcium phosphate, as used in the initial HTO of our patient, is osteoconductive and has less compressive strength than calcium phosphate. However, its compressive strength is similar to cancellous bone [15].

The choice of the plate is debatable as well. In the study of Lash et al. comparing TomoFix plate (locking screw fixation, DePuySynthes, USA) and Puddu® plate (nonlocking plate, Arthrex, USA), the risk of delayed union or nonunion is slightly lower in the TomoFix group, with 2.6% of occurrences versus 3.7%, respectively [16]. Nevertheless, these results were not statistically significant. This tendency is found in other studies [17], including biomechanical studies [18]. However, other authors, such as Woodacre et al. did not find any correlation between the plate and union rates [14]. In our case, the TomoFix plate was used in the 2 surgical interventions.

It can be asked whether the infection in 2013 is a cellulitis, complicated with infection at the nonunion site extended to septic knee arthritis or a resurgence of a low-grade infected HTO. As no tissue sample was analyzed at the early postoperative infection in 2009, we cannot state that this is not a resurgence of a low-grade infection from 2009. A low-grade infection could explain the nonunion, despite the revision in 2010. However, our patient has also other risk factors that could explain the nonunion, without persistent infection involved. The overweight put her at a higher risk of a loss of correction. Furthermore, the broken lateral cortex of the proximal tibia increases the local instability and the risk of nonunion.

Concerning the treatment of the infected nonunion complicated with septic arthritis, we decided to treat it with a 2-stage procedure, as we would do for a chronic, infected, total knee prosthesis. After resection of the partially avascular, infected articular block and wide debridement, we implanted a nonarticulating, antibiotic-loaded cement spacer and treated the patient with intravenous antibiotics. This was followed after a short interval (of 3 weeks) by an implantation of a hinged, total knee arthroplasty.

For our patient, another attempt of osteosynthesis was not an option, as septic arthritis had destroyed the remaining cartilage and had severely worsened the preexisting gonarthritis. In this situation, a total knee replacement needs to be considered as a valid option. However, the infection associated with nonunion would make a one-stage procedure hazardous. So, infection was treated first with the removal of the infected bone and the soft tissues, and then a spacer was implanted to avoid retraction of the soft tissues until prosthesis implantation. Local antibiotics were mixed with the cement for local treatment (gentamicin, tobramycin, and vancomycin). A nonarticulating spacer was implanted to avoid tension on the bad quality soft tissues and enable the patient to walk with partial weight bearing.

In our hospital, if a 2-stage exchange is used, we do not do the free interval without antibiotics before reimplantation of prosthesis and we consider the alternatives of short and long intervals of time, depending on the soft tissue situation, the bacteria found on the microbial work up, and their resistance patterns. In our case, as the bacteria could be identified by PCR, and was an easy-to-treat germ, we were able to choose a short interval and implant the prosthesis after only 3 weeks.

Finally, the implant chosen was a stemmed, rotating hinge prosthesis, considering the bony defect and the associated loss of concomitant ligamentous structures [19] combined with the obesity of the patient [20].

In the literature, cohort studies are missing concerning the treatment of infected nonunions after HTO. Current data are based mostly on case reports, as this pathology is quite rare. Indeed, the infection rate following HTO is estimated at 3.6–4.5% [21, 22]. We could not find any data evaluating septic nonunion of HTO.

Management of infected, high tibial osteotomy differs, depending on the stage of infection. Usually, the first line of treatment includes debridement and antibiotics and is usually followed by hardware removal [22–24]. Nevertheless, when infection appears as an early complication, before consolidation of the site of osteotomy, hardware removal may be problematic, as it causes loss of correction and loss of benefit of the HTO. In this case, Chae [25] proposed plate removal, curettage, and filling the bone defect at the HTO site with cement containing antibiotics. But this study showed a 30% to 40% loss of alignment on the HTO site.

In infected nonunion HTO where gonarthritis could still be treated conservatively, different types of reosteosynthesis have been recommended. In a retrospective study, Ring et al. [26] compared treatment with an Ilizarov external fixator, versus plate osteosynthesis and autologous bone graft, following bone debridement. Both techniques have proven efficient, but external fixation had a higher complication rate with a longer duration of treatment and a nonnegligible risk of pin infection.

Seki et al. [7] successfully treated one case with hardware removal and filling of the osteotomy defect with vancomycin-impregnated, calcium phosphate cement associated with external fixation for 12 months and concomitant antibiotics for 3 months.

McHale et al. [8] showed another variant with gentamicin-impregnated PMMA cement and an Ilizarov external fixator. Nevertheless, PMMA needs to be removed once the infection is cured and replaced by a bone graft to treat the pseudarthrosis.

When gonarthritis associated with nonunion HTO was too severe for osteosynthesis, some authors recommended total knee replacement [27, 28]. Yoshino et al. [27] chose a graft iliac crest bone and waste of femoral cut to fill the bone defect at the site of nonunion and stabilized it with a long-stem TKA. In another case report, Gandhi et al. [28] implanted a posterior stabilized TKA with long stem passing through osteotomy to reinforce stability.

Finally, only one paper presented 7 cases with a 2-stage procedure in nonunion HTO combined with infection. Karatosun et al. [6] proposed large debridement and implant removal but with no antibiotic spacer. Once the blood inflammatory parameters were normalized, a total knee replacement was implanted. In the interval, the knee was immobilized in extension in a splint and the patient could walk with crutches and no weight bearing.

Infected nonunion after HTO is a rare complication. As in any surgery, the first step is to select the right operative indication. In obese patients, we saw that, although there is a theoretical relative contraindication, HTO is a debatable but appropriate surgical option. Unfortunately, this category of patient has higher risks of loss of correction and lower functional outcome. However, with the rising incidence of overweight patients, this issue is likely to increase in the future.

Once an infection has occurred in a patient, the most important step is to well document the bacteria plus its susceptibility to antibiotics and then discuss the case with an infectious disease specialist.

In septic orthopaedic surgery, the multidisciplinary approach with an infectious disease specialist (trained in the treatment of foreign body infections) is of major importance.

In our case, data of the early postoperative infection were incomplete. However, this situation is not that uncommon in the clinical practice. These missing data did not compromise the evolution of the final total knee replacement, as the patient was free of infection 2 years after the two-stage implantation.

Once nonunion and infection are identified, we must develop a plan to treat both the instability and the infection. As mentioned above, different options exist based on this same principle: eradication of the infection and stabilization. Our case proves that when a tricompartmental arthritis has occurred, infected nonunion is not a contraindication for implantation of a prosthesis.

## 4. Conclusion

When infection is suspected after HTO, blind antibiotics without diagnosis should never been given to a patient. Surgical treatment in these situations is mandatory, for the removal of all foreign bodies as is adapted local and systemic antibiotic treatment. If the first revision leads to successful healing of the HTO, early removal of the hardware can be performed. In cases of ongoing infection and progressive joint destruction, a 2-stage total joint arthroplasty is a valid option.
